# Genotoxicity of Copper and Nickel Nanoparticles in Somatic Cells of* Drosophila melanogaster*

**DOI:** 10.1155/2018/7278036

**Published:** 2018-07-19

**Authors:** Erico R. Carmona, Alba García-Rodríguez, Ricard Marcos

**Affiliations:** ^1^Núcleo de Investigación en Bioproductos y Materiales Avanzados (BioMA), Facultad de Ingeniería, Universidad Católica de Temuco, Chile; ^2^Departamento de Procesos Industriales, Facultad de Ingeniería, Universidad Católica de Temuco, Chile; ^3^Grup de Mutagènesi, Departament de Genètica i de Microbiologia, Facultat de Biociències, Universitat Autònoma de Barcelona, Cerdanyola del Vallès (Barcelona), Spain; ^4^CIBER Epidemiología y Salud Pública, Instituto de Salud Carlos III, Madrid, Spain

## Abstract

Copper and nickel nanoparticles (Cu-NPs and Ni-NPs, respectively) are used in a variety of industrial applications, such as semiconductors, catalysts, sensors, and antimicrobial agents. Although studies on its potential genotoxicity already exist, few of them report* in vivo* data. In the present study we have used the wing-spot assay in* Drosophila melanogaster* to determine the genotoxic activity of Cu-NPs and Ni-NPs, and these data have been compared with those obtained with their microparticle forms (MPs). Additionally, a complete physical characterization of NPs using transmission electronic microscopy (TEM), dynamic light scattering (DLS), and laser Doppler velocimetry (LDV) techniques was also performed. Results obtained with Cu-NPs and Cu-MPs indicate that both failed to induce an increase in the frequency of mutant spots formation in the wings of the adults, suggesting a lack of genotoxicity in somatic cells of* D. melanogaster*. However, when Ni-NPs and Ni-MPs were evaluated, a significant increase of small single spots and total mutant spots was observed only for Ni-NPs (P<0.05) at the highest dose assessed. Thus, the genotoxicity of Ni-NPs seem to be related to their nanoscale size, because no genotoxic effects have been reported with their microparticles and ions. This study is the first assessing the* in vivo* genotoxic potential of Cu-NPs and Ni-NPs in the* Drosophila* model.

## 1. Introduction

Given their particular physical-chemical features and low cost of fabrication, copper and nickel nanoparticles (Cu-NPs and Ni-NPs, respectively) are being widely used in different industrial applications. On the one hand, Cu-NPs show high thermal and electrical conductivity and are being used in the manufacture of lubricants, polymers, plastics, metallic coatings, and electronic devices. Additionally, they have shown antimicrobial properties, and therefore Cu-NPs have been used as potential antibacterial and/or antifungal agents [1]. On the other hand, Ni-NPs have high electrical conductivity and resistance to corrosion, being commonly used in medical devices, sealants, plastics, and electronic equipment [2]. In spite of the growing use of both metallic NPs, information about their toxic and genotoxic effects is limited.

The extremely small size of NPs allows reaching easily the nucleus of live cells through penetration via nuclear pore or during mitosis and interacting directly with DNA organized in chromatin or chromosomes causing genetic damage [3]. However, to induce DNA damage, NPs do not need to be in contact with DNA because NPs can interact with proteins involved in DNA replication, mitotic division, and also generate high quantities of oxidative stress, which can induce indirect damage to DNA [3]. Thus, the genotoxic effects of NPs should be evaluated as they potentially can generate and contribute to human and environmental health risks.

From the available literature, we found some* in vitro* studies that suggest that Cu-NPs induced cell death and viability loss in neurons, suggesting a potential neurotoxicity and neurodegeneration mechanism associated [4, 5].

Instead,* in vivo* studies carried out with mice showed that Cu-NPs can induce several toxicological effects and injuries on kidney, liver, and spleen, but microparticles or bulk forms of copper do not [6]. The above have been observed also in fish organisms where Cu-NPs have demonstrated that they are acutely toxic to zebrafish, and also Cu-NPs produced different morphological effects and global gene expression patterns in the zebrafish gills, but soluble copper do not show the same effects, suggesting a higher toxicity effect of these metallic NPs [7]. Recently, Cu-NPs were found mutagenic in the Ames bacterial reverse mutation assay; also these can induce significant increase of binucleated cells with micronuclei at the highest concentration and can promote DNA strand breaks and oxidative DNA damage, suggesting a genotoxic risk associated with Cu-NPs exposure [8].

Ni-NPs are undoubtedly the less studied among other metallic NPs. However, there is also some evidence that Ni-NPs may induce oxidative stress and apoptosis in human cells [9]. In addition, Ni-NPs could also promote sarcoma and activate or upregulate genes in pathways related to cancer [10], and in* in vitro* studies both metallic nickel fine and nanoparticles can induce high carcinogenic potential in mouse epidermal JB6 cells [11]. Although there is a lack of* in vitro* and* in vivo* genotoxic and mutagenic studies for both metallic nickel and nickel-based NPs, recent studies indicate that Ni-NPs had higher cytotoxic and genotoxic effects than Ni microparticles in* in vitro* and in* in vivo* studies under the same treatment doses using human cells and rat models [12]. It should be noted that recent studies indicate that nickel oxide-NPs are genotoxic and mutagenic in mammals cells* in vitro* and in* Drosophila melanogaster* model [13].

In the present study, the* in vivo* mutagenic and recombinogenic activity associated with the exposure of Cu-NPs and Ni-NPs measured by the wing-spot assay in* Drosophila melanogaster* was assessed. It is important to note that in recent years the fruit fly* D. melanogaster* has played a relevant role in detecting* in vivo* genotoxic and mutagenic action of several kinds of metal and metal-oxides NPs, offering several biological and genetic advantages to study acute and chronic effects and the underlying mechanisms of NPs action [14].

## 2. Materials and Methods

### 2.1. Drosophila Strains

The following mutant* Drosophila *strains were used for the wing-spot test: the* multiple wing hairs* strain with the genetic constitution* y*;* mwhj*; the* flare-3* strain with the genetic constitution* flr*^*3*^/*ln* (*3LR*)* TM3*,* Bd*^*s*^. The* multiple wing hairs marker* (*mwh*, 3–0.3) is a completely recessive homozygous viable mutation, which is kept in homozygous condition. It produces multiple trichomes per cell instead of the normally unique trichome in the wing cells. The* flare-3* marker (*flr*^*3*^, 3–38.8) is a recessive mutation that affects the shape of wing hairs, producing malformed wing hairs that have a flare shape. Given their zygotic lethality,* flare* alleles have to be kept in stocks over balancer chromosomes carrying multiple inversions and a dominant marker that is a lethal homozygous (*TM3*,* Bd*^*s*^). More detailed information on genetic markers and descriptions of the phenotypes are given by Lindsley and Zimm [15]. Both strains were cultured in glass bottles with the standard medium for* Drosophila* (i.e., agar, corn flour and yeast) at a temperature of 25 ± 1°C and a relative humidity of ~60%.

### 2.2. Chemicals

Copper nanoparticles (Cu-NPs, 60-80 nm, CAS 7440-50-8, Ref. 774103) and nickel nanoparticles (Ni-NPs, <100 nm, CAS 7440-02-0) were purchased from Sigma-Aldrich (St Louis, USA). Copper microparticles (Cu-MPs, <63 *μ*m, CAS 7440-50-8, Ref. 102703) and nickel microparticles (Ni-MPs, 10 *μ*m, 7440-02-0, Ref. 112277) were obtained from Merck Company (Darmstadt, Germany).

### 2.3. Characterization of NPs

Due to the fact that detailed information provided by chemical suppliers about equipment and methodologies used for physical characterization of NPs are restricted, an exhaustive physical characterization of Cu and Ni-NPs was carried out in the present study. For this, transmission electron microscopy (TEM), dynamic light scattering (DLS), and laser Doppler velocimetry (LDV). TEM were carried out with a JEOLJEM- 2011 instrument to determine the size of NPs in dry form. DLS and LDV were performed with a Malvern Zetasizer Nano-ZS zen3600 instrument to measure the hydrodynamic diameter and zeta potential in aqueous suspension, respectively. For TEM analyses, metal-NPs were measured at a concentration of 2.56 mg/mL. For DLS and LDV techniques, NPs samples were measured at a concentration of 10 *μ*g/mL.

### 2.4. Particles Preparation

To particle preparation, we followed the methods of Carmona et al. [16] where various concentrations of Cu-NPs and Ni-NPs (from 1 mM to 10 mM) were prepared and diluted with distilled water with magnetic stirring. After that, dispersion was carried out by sonication using an ultrasonic bath (Elmasonic S, 37 KHz) for 30 min at room temperature. Cu-MPs and Ni-MPs were used to compare the genotoxic effects between microparticle and nanoparticulated forms. These compounds were prepared with distilled water and diluted through a magnetic stirring for 10 min at room temperature. Distilled water was used as negative control, while the mutagenic agent Ethyl methane sulphonate (EMS) at 1 mM was used as positive control in each experiment carried out with the wing-spot.

### 2.5. Toxicity of Particles in* Drosophila*

The different concentrations of nano- and microparticles of Cu and Ni used in the genotoxicity experiments with* D. melanogaster *strains were selected according to previous toxicity and viability studies carried out (data not shown). On the one hand, a range of doses from 0.1 to 10 mM was established for both particle sizes of copper, and within these dose range Cu-NPs showed higher toxicity than Cu-MPs. Hence, while for Cu-MPs a suitable larval viability (>70%) was reached at 10 mM, Cu-NPs could not be evaluated at concentration higher than 5 mM.

On the other hand, a range of doses from 1 to 10 mM was selected for Ni particles, and within this range a suitable viability was obtained (>70%). In general, both Ni-NPs and Ni-MPs showed similar toxicity levels; therefore both particles were assayed in the same concentrations. The main criterion to choose the final concentrations was the number of emerging larvae and adults after treatments were high enough to perform genotoxic experiments with the wing-spot test [17].

### 2.6. Wing-Spot Tests Protocol

The wing-spot test was used as a short test system based on the loss of heterozygosity (LOH) in normal genes and the corresponding expression of recessive markers, called* multiple wing hairs* (*mwh*) and* flare-3* (*flr*^*3*^), in the wing blade of adult flies [18]. Thus, the induced genotoxic effects are microscopically observed as an increase in the frequency of mutant clones cells (*mwh* or* flr*^*3*^ phenotype) in wing slides preparations. This assay can detect a wide range of mutational events such as point mutations, deletions, certain types of chromosome aberrations (nondisjunction), and somatic recombination [18].

Virgin females of the* flr*^*3*^ strain were mated to* mwh* males as previously described [17]. Eggs from this cross were collected during 8 h periods in culture bottles containing the standard medium. The resulting 3-day-old larvae (third instar larvae) were then placed in plastic vials containing 4.5 g of* Drosophila* instant medium (Carolina Biological Supply, Burlington, NC) prepared with 10 mL of various nontoxic concentrations of Cu-NPs (0.1, 1, and 5 mM), Cu-MPs, Ni-NPs, and Ni-MPs (1, 5, and 10 mM). Larvae were fed in this medium until pupation. The surviving adults were collected and stored in 70% ethanol. Afterwards, their wings were removed with fine tweezers and mounted in Faure's solution on microscope slides. The wings were scored at 400 times magnification for the presence of small single spots, large single spots, and twin spots. Small single* flr*^*3*^ spots also were scored in the wing samples, but these were included in the total mutant spots, as has been usually expressed in previous works [19]. In each series, 80 wings were scored (from 40 individuals). Scoring of flies and data evaluation were conducted following the standard procedures for the wing-spot test, as used in recent investigations [20].

### 2.7. Statistical Analysis

The conditional binomial test was applied to assess differences between the frequencies of each type of spot in treated and concurrent negative control with significant levels *α* = *β* = 0.05 [21]. The multiple-decision procedure was used to judge the overall response of an agent as positive, negative, or inconclusive [22]. The treatment was considered as positive if the frequency of mutant clones in the treated series was at least* m* (multiplication factor) times greater than in the control series. Since small single spots and total spots have a comparatively high spontaneous frequency,* m* was fixed at a value of 2 (testing for a doubling of the spontaneous frequency). For large single spots and twin spots, which have a low spontaneous frequency,* m* = 5 was used. The frequency of clone formation was calculated, without size correction, by dividing the number of* mwh* clones per wing by 24,400, which is the approximate number of cells inspected in one wing [23].

## 3. Results and Discussion

In order to confirm the physical characteristics of metallic NPs given by the supplier TEM, DLS and LDV techniques were performed to assess diameter, shape, hydrodynamic size, and stability of Cu-NPs and Ni-NPs, respectively.

**Figure 1:**
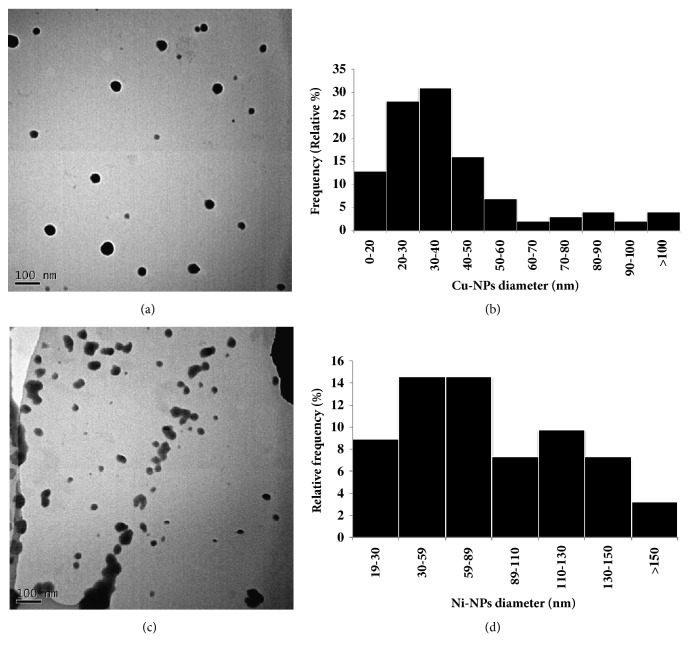
Physical characterization of metal-NPs with TEM. (a) and (c) are images with high magnification of Cu-NPs and Ni-NPs, respectively (bar scales representing 100 nm). (b) and (d) are bar charts showing size distribution of Cu-NPs and Ni-NPs, respectively.

TEM was used to characterize dry size of metal-NPs. On the one hand, Cu-NPs displayed spherical shapes and showed low levels of agglomeration (Figure 1(a)). The size of the Cu-NPs ranged from 9.6 to 100 nm diameter, and the average (±SD) diameter was 33.34 ± 14.81 nm (Figure 1(b)). TEM images and analyses of representative Cu-NPs (n = 100) indicated that size was lesser than from the manufacturer's indications (60-80 nm).

On the other hand, Ni-NPs exhibited mainly oval shapes and their NPs showed a certain level of agglomeration (Figure 1(c)). The size of Ni-NPs ranged from 19.30 to 187.27 nm diameters, and the average (±SD) was 76.65 ± 45.38 nm (Figure 1(d)). TEM analysis of Ni-NPs indicated that the size was similar to the manufacture's indications (<100 nm).

Hydrodynamic characterization of metal-NPs was summarized in Table 1. The diameter average of metal-NPs in water suspension was different and higher than TEM analyses, reaching the mean value of 260.9 nm for Cu-NPs and 217.5 nm for Ni-NPs. These differences are commonly explained by the tendency of NPs to agglomerate in aqueous medium, making them larger than in primary size [24]. The average of zeta potential was −19.6 mV for Cu-NPs and −24.6 mV for Ni-NPs, indicating a moderate stability and dispersion of these nanocompounds in aqueous medium for feeding* D. melanogaster* larvae [25].

The data of Cu-NPs and Cu-MPs from the wing-spot test was summarized in Table 2. Although the frequency of clone formation increases with a dependent concentration manner (from 1.35 to 2.01), the results indicate that Cu-NPs do not induce significant increases in the frequency of any mutant spots, as compared with the negative control. In addition, Cu-MPs showed similar negative results, suggesting that both particles do not promote mutation events and recombination activity in* Drosophila*.

Table 3 summarizes the results obtained with Ni-NPs and Ni-MPs treatments. The results indicate that Ni-NPs can induce significant increases in the frequency of single small spots and the total mutant spots. The above was confirmed also with the frequency of clone formation, which showed a clear increase tendency when concentrations of Ni-NPs increase (1.15 to 2.50). However, Ni-MPs do not induce significant increases of mutation spots, suggesting that NPs could be responsible for the genotoxic effects observed. It should be noted that the averaged of mutant spot frequencies recorded in the negative controls (0.28–0.41) were in accordance with the normal background range observed in the laboratory and are not significantly different from previous results [26]. The positive controls carried out with 1 mM EMS showed a clear response, and the mutant spot frequencies also agreed with previous and recent studies.

In present study, the wing-spot test of* Drosophila* was chosen to evaluate genotoxicity of copper and nickel nanomaterials. This* in vivo* short-term test using a eukaryotic organism is a comprehensive genotoxic assay as diverse mutational events can be measured, such as point mutations, deletions, and certain types of chromosome aberrations. Besides, the wing-spot assay also distinguishes mitotic recombination in proliferative somatic cells, being the quantitation of the recombinogenic activity of a compound of primary importance for genotoxicity screening due to the strong relationship with carcinogenic process [27, 28]. Thus, this assay offers many advantages and it has proved to be an excellent candidate to be used as biological monitor for genotoxic nanomaterials [14].

In general, there are few genotoxic and mutagenic studies about Cu-NPs available in the literature, which are possibly difficult to be compared with the present study in* Drosophila*.

However,* in vitro* studies indicate that Cu-NPs can induce cell death, viability loss, dopamine depletion, alteration of dopaminergic gene system expression, and oxidative stress in neurons of rats, suggesting a potential neurotoxicity and neurodegeneration mechanism associated with the acute exposition of Cu-NPs with average diameter between 40 and 90 nm [4, 5]. In addition, Cu-NPs can induce several toxicological effects and injuries on kidney, liver, and spleen in mice exposed to Cu-NPs with average diameter of 24 nm [6]. In zebrafish models (*Danio rerio*), Cu-NPs with average size of 80 nm have showed that they were acutely toxic to zebrafish at low concentration (1.5 mg/L), while nontoxic concentration (100 *μ*g/L) can induce gill injury, affect biochemical markers of gill function, and alter stress and metal responsive gene expression in their gills [7]. Thus, these kinds of metallic NPs seem to induce toxic effects in different test system including mammals and fish organisms.

Until now, only a single study had been published about genotoxicity of Cu-NPs. In this study, Cu-NPs were found to be mutagenic in the Ames test (TA98 and TA100 bacterial strains) and also found toxic for bacteria cells (cytotoxicity) in dose-dependent manner. Also, Cu-NPs induced significant increase in number of binucleated cells with micronuclei in monkey kidney cells at the highest concentration evaluated, suggesting chromosome damage* in vitro*. Finally, Cu-NPs also induced DNA strand breaks measured by comet assay and oxidative DNA damage associated [8]. These results contrast with those observed in the wing-spot assay, where pure Cu-NPs did not promote mutation and recombination activity in somatic cells of* D. melanogaster*. But these contrasts can be given by the use of different system assays (*in vitro* versus* in vivo* tests) and organisms models (bacteria and mammals versus insects) for genotoxic screening. Thus, further comprehensive studies including different and combination of bioassays for toxic and genotoxic assessment are requested to clarify genetic effects of Cu-NPs; especially* in vivo* studies are needed to identify mechanisms of genotoxicity in whole organisms.

On the other hand, Cu-NPs showed high hydrodynamic size (261 nm) and low Z potential, indicating agglomeration and low stability of particles in aqueous medium, which represent the real conditions of NPs exposure in experiments with SMART test of* Drosophila*. Thus, the absence of genotoxicity found in this study could be related to the change of physical properties of Cu-NPs losing biological reactivity (given by small size and great surface area) and the capacity of generate DNA damage by direct or indirect mechanisms in somatic cells of* D. melanogaster. *However, the absence of positive results with Cu-NPs in the wing-spot test could be related also to their poor solubility of this nanomaterial in water media, because previously SMART studies indicated positive results with a more soluble form of copper: CuO-NPs [26]. Thus, the Cu ions released from NPs can be considered one the main mechanisms of toxicity on living cells, suggesting that the relation between solubility and genotoxicity of NPs is an aspect to be accounted for when interpreting nanotoxicology results from Cu-NPs [29].

Although few data about genotoxic effects of single Ni-NPs are available in the literature, the results obtained in this research are in agreement with recent studies carried out in* in vitro* and* in vivo* studies. Evidences from DNA fragmentation analysis using the comet assay showed that the Ni-NPs with average diameter of 52 nm cause primary DNA damage in a dose- and time-dependent manner. These Ni-NPs were also found to induce oxidative stress evidenced by the generation of reactive oxygen species (ROS) and depletion of glutathione (GSH), suggesting the capability of the Ni-NPs to induce oxidative stress resulting in genotoxicity for human skin cells (A431) [30]. Other nickel-based nanomaterials such as nickel oxide nanoparticles (NiO-NPs) have shown to induce cell cycle alteration in cell lines of human pulmonary epithelial (BEAS-2B and A549) even in different phases and these modifications may be induced by the NPs genotoxic effect, suggested by the nuclear translocation of phospho-ATM and phospho-ATR [31].

On the other hand,* in vivo* studies carried with female Wistar rats assessing genotoxicity with micronucleus, chromosome aberrations, and the comet assays indicate that the NiO-NPs administered through the oral route were capable of inducing chromosome alterations and DNA breaks at the high-dose level and these effects were more prominent at 24 h sampling time [32].

Besides the genotoxic effects, it should be noted that Ni-NPs have shown to induce effects on tumour promoter or suppressor gene expressions as well as on cell transformation in mouse epidermis cells (JB6 cells), suggesting that metallic Ni-NPs can be carcinogenetic* in vitro* [11]. Thus, the use of metallic Ni-NPs for future application should be taken with caution due to their toxicity and carcinogenicity potential.

Our results report for the first time data on the evaluation of Ni-NPs genotoxicity with the* in vivo* wing-spot test of* Drosophila*. Treatments with high concentrations of Ni-NPs induce a significant increase on the frequency of single and total of spots analysed; however, Ni-MPs were negative to induce mutant spots. Due to the fact that Ni-NPs mainly induce single mutant and low number of twin spots, the genotoxicity observed for Ni-NPs can be attributed mainly for mutational events (i.e., point mutation, deletion and chromosome disjunction), not including recombinogenic activity. Considering that previous studies with ionic and soluble forms of nickel (e.g., NiCl_2_ and NiSO_4_) have shown negative results in the* Drosophila* wing-spot test [33], the genotoxic effects found with Ni-NPs indicate that the intrinsic nanoscale size and large surface area properties of these particles play a relevant role in the induction of somatic mutation in* Drosophila*. In addition, recent studies with NiO-NPs also indicate genotoxic and mutagenic in* Drosophila* with this kind of metallic nanoparticles supporting the genotoxic effects found in the present study [13]. Although oxidative DNA damage was not evaluated in this study with* Drosophila*, previous studies have demonstrated that Ni-NPs can generate intracellular oxidative stress damaging DNA in cells, suggesting that this mechanism of action is responsible for their genotoxicity [8, 12, 34].

## 4. Conclusion

Cu-NPs failed to induce an increase of mutant spots in the wings of fruit flies adults, suggesting a lack of genotoxicity in somatic cells of* D. melanogaster*. However, when Ni-NPs were evaluated, a significant increase of small single spots and total mutant spots was observed at the highest dose assessed. Thus, the genotoxicity of Ni-NPs seems to be related to their nanoscale size, because no genotoxic effects have been reported with their microparticles and ion forms. This study was the first assessing the* in vivo* genotoxic potential of Cu-NPs and Ni-NPs in the* Drosophila* model.

## Figures and Tables

**Table 1:** Physical characterisation of metal-NPs by DLS and LDV methodologies. Values indicate mean and ± standard deviation for each parameter.

NPs	Hydrodynamic diameter (nm)	Z potential (mV)	Mob (*μ*mcm/Vs)
Cu	260.9 ± 8.29	-19.6 ± 0.5	-1.53 ± 0.04
Ni	217.5 ± 10.25	-24.6 ± 2.20	-1.51 ± 0.12

**Table 2:** Genotoxicity data obtained from the *Drosophila* wing-spot test experiments with copper nanoparticles (CuNPs) and microparticles (CuMPs). Results from *mwh/flr*^*3*^ wings.

Compound, concentration (mM)	Small single spots (1-2 cells) (*m* = 2)			Large single spots (>2 cells) (*m* = 5)			Twin spots (*m* = 5)			Total spots (*m* = 2)			Frequency of clone formation per 10^5^ cells
	No	Fr	D	No	Fr	D	No	Fr	D	No	Fr	D	
**CuNPs**													
Control	22	0.28		3	0.04		1	0.01		26	0.33		1.35
0.1	29	0.36	i	5	0.06	i	1	0.01	i	35	0.44	i	1.80
1	26	0.33	-	4	0.05	i	3	0.04	i	33	0.41	i	1.68
5	34	0.43	i	5	0.06	i	0	0.00		39	0.49	i	2.01
EMS													
1	205	2.56	+	59	0.74	+	41	0.51	+	337	4.21	+	17.25

**CuMPs**													
Control	21	0.26		2	0.03		1	0.01		24	0.30		1.23
1	26	0.33	i	1	0.01	i	3	0.04	i	30	0.38	i	1.56
5	30	0.38	i	2	0.03	i	3	0.04	i	35	0.44	i	1.80
10	30	0.38	i	0	0.00		4	0.05	i	34	0.43	i	1.76
EMS													
1	227	2.84	+	37	0.46	+	40	0.50	+	322	4.03	+	16.52

No: number of spots, Fr: frequency, D: statistical diagnosis, +: positive, -: negative, i: inconclusive, *m*: multiplication factor, probability levels, *α* = *β* = 0.05; 80 wings were analysed for each concentration (40 individuals).

**Table 3:** Genotoxicity data obtained from the *Drosophila* wing-spot test experiments with nickel nanoparticles (NiNPs) and microparticles (NiMPs). Results from *mwh/flr*^*3*^ wings.

Compound, concentration (mM)	Small single spots (1-2 cells) (*m* = 2)			Large single spots (>2 cells) (*m* = 5)			Twin spots (*m* = 5)			Total spots (*m* = 2)			Frequency of clone formation per 10^5^ cells
	No	Fr	D	No	Fr	D	No	Fr	D	No	Fr	D	
**NiNPs**													
Control	20	0.25		1	0.01		1	0.01		22	0.28		1.15
1	26	0.33	i	2	0.03	i	0	0.00		28	0.35	i	1.43
5	32	0.40	i	1	0.01	i	1	0.01	i	34	0.43	i	1.76
10	43	0.54	+	5	0.06	i	1	0.01	i	49	0.61	+	2.50
EMS													
1	202	2.53	+	57	0.71	+	40	0.50	+	321	4.01	+	16.43

**NiMPs**													
Control	30	0.38		2	0.03		1	0.01		33	0.41		1.68
1	35	0.44	-	1	0.01	i	2	0.03	-	38	0.48	-	1.97
5	26	0.33	-	4	0.05	i	0	0.00		31	0.39	i	1.60
10	25	0.31	-	2	0.03	i	1	0.01	i	29	0.36	i	1.47
EMS													
1	170	2.13	+	31	0.39	+	26	0.33	+	245	3.06	+	12.54

No: number of spots, Fr: frequency, D: statistical diagnosis, +: positive, -: negative, i: inconclusive, *m*: multiplication factor, probability levels, *α* = *β* = 0.05; 80 wings were analysed for each concentration (40 individuals).

## Data Availability

The data used to support the findings of this study are included within the article.
